# Fall incidents unraveled: a series of 26 video-based real-life fall events in three frail older persons

**DOI:** 10.1186/1471-2318-13-103

**Published:** 2013-10-04

**Authors:** Ellen Vlaeyen, Mieke Deschodt, Glen Debard, Eddy Dejaeger, Steven Boonen, Toon Goedemé, Bart Vanrumste, Koen Milisen

**Affiliations:** 1 Center for Health Services and Nursing Research, Faculty of Medicine, KU Leuven, Kapucijnenvoer 35/4, 3000 Leuven, Belgium; 2 Division of Geriatric Medicine, Leuven University Hospital, Herestraat 49, 3000 Leuven, Belgium; 3 MOBILAB, Biosciences and Technology Department, Thomas More Kempen, Kleinhoefstraat 4, 2440 Geel, Belgium; 4 Center for Processing Speech and Images, Department of Electrical Engineering, KU Leuven, Kasteelpark Arenberg 10, box 2440, 3001 Heverlee, Belgium; 5 Center for Metabolic Bone Diseases, KU Leuven, Herestraat 49, box 701, 3000 Leuven, Belgium; 6 Campus De Nayer, Lessius Hogeschool, Jan De Nayerlaan 5, 2860 Sint-Katelijne-Waver, Belgium; 7 iMinds Future Health Department/SISTA/SCD/ESAT, KU Leuven, Kasteelpark Arenberg 10, postbus 2440, 3001 Heverlee, Belgium

**Keywords:** Accidental falls, Detection, Fall characteristics, Older persons, Video-based, Classification system

## Abstract

**Background:**

For prevention and detection of falls, it is essential to unravel the way in which older people fall. This study aims to provide a description of video-based real-life fall events and to examine real-life falls using the classification system by Noury and colleagues, which divides a fall into four phases (the prefall, critical, postfall and recovery phase).

**Methods:**

Observational study of three older persons at high risk for falls, residing in assisted living or residential care facilities: a camera system was installed in each participant’s room covering all areas, using a centralized PC platform in combination with standard Internet Protocol (IP) cameras. After a fall, two independent researchers analyzed recorded images using the camera position with the clearest viewpoint.

**Results:**

A total of 30 falls occurred of which 26 were recorded on camera over 17 months. Most falls happened in the morning or evening (62%), when no other persons were present (88%). Participants mainly fell backward (initial fall direction and landing configuration) on the pelvis or torso and none could get up unaided. In cases where a call alarm was used (54%), an average of 70 seconds (SD=64; range 15–224) was needed to call for help. Staff responded to the call after an average of eight minutes (SD=8.4; range 2–33). Mean time on the ground was 28 minutes (SD=25.4; range 2–59) without using a call alarm compared to 11 minutes (SD=9.2; range 3–38) when using a call alarm (p=0.445).

The real life falls were comparable with the prefall and recovery phase of Noury’s classification system. The critical phase, however, showed a prolonged duration in all falls. We suggest distinguishing two separate phases: a prolonged loss of balance phase and the actual descending phase after failure to recover balance, resulting in the impact of the body on the ground. In contrast to the theoretical description, the postfall phase was not typically characterized by inactivity; this depended on the individual.

**Conclusions:**

This study contributes to a better understanding of the fall process in private areas of assisted living and residential care settings in older persons at high risk for falls.

## Background

Fall incidents and their consequences are a significant threat to older people’s health. In the community setting falls occur frequently: 28 to 35% of older people fall at least once every year, 25% of these fallers fall more than once [1,2]. In residential care facilities, the average fall incidence is three times higher than in the community [3]. Falls often lead to various injuries ranging in severity from bruises and lacerations to brain trauma and fractures [4,5]. Additionally, falls have serious psychological consequences (e.g. fear of falling) and result in a significant economic burden [6,7].

Depending on the study population, 20 to 43% of community-dwelling older persons who fall are not able to get up unaided [8-10]. In sheltered housing and institutional settings 66 to 100% of residents need help to get up [8]. Lying on the ground too long increases the risk of negative outcomes, such as dehydration, hypothermia, pressure ulcers, bronchopneumonia and death [8,9]. Although there is evidence that unifactorial (e.g. exercise program) and multifactorial (e.g. falls risk assessment and management of identified fall risk factors) preventive approaches can reduce falls in older persons, it is unlikely for any strategy to prevent every single fall incident [11,12].

In case fall prevention fails, other interventions are needed to reduce the clinical risks associated with lying on the ground too long. A potentially appealing option is a camera-based fall detection system. Our research project ‘FallCam: camera system for fall detection in older persons’ was set up to build a prototype camera system, designed to detect falls [13,14]. To detect falls and validate the camera system, it is essential to unravel the way in which older people fall. However, this process is difficult to describe. Noury et al. [15] proposed a classification system that divided a fall into four phases; the prefall, critical, postfall and recovery phase. However, the description of falls is mostly based on retrospective methods (e.g. interview), and the accuracy of this approach remains uncertain [16]. Moreover, older persons, especially those with cognitive impairment, often do not recall falls [17]. Obtaining information from real-life images may therefore be critical for developing a fall detection system. In addition, information on activities associated with falls, balance recovery or protective responses such as stepping or grasping and impact to key body sites is important for future fall and injury prevention [16,18,19].

To the authors’ knowledge, only one study provided a description of the cause and activity at the time of falling of video-based real-life falls [20]. However, video cameras were installed in common areas of long-term care facilities (e.g. dining rooms, lounges, hallways) and not in the participant’s person living space, as the current study did, which presents a different environmental and situational context.

The aim of this study was to provide a thorough analysis and description of video-based real-life fall events and to examine the phases of real-life falls using the classification system suggested by Noury et al. [15].

## Methods

### Design and participants

An observational study of three older persons was conducted from July 2009 to April 2010 in two settings (assisted living and residential care, respectively) in Belgium. Because of the profound nature of the study, recruitment of eligible persons was done via the staff of the facility, who had an interpersonal trust relationship with the residents. For that reason, information on the number of residents approached to test the surveillance system and numbers on refusal are unknown. The target population were individuals aged 65 years or older with a high risk of falling, which was defined as a minimum of one fall in the last six months and/or having difficulties with gait and balance. Potential candidates received oral and written information concerning the aims of the study, and any positive and negative aspects of participation. If the person showed interest, the staff contacted the research team so eligibility of each person could be assessed and more detailed information about the study could be provided. Residents provided written informed consent to participate in the study. If residents were unable to do so, a relative would provide written consent.

### Procedures and measurements

**Figure 1:**
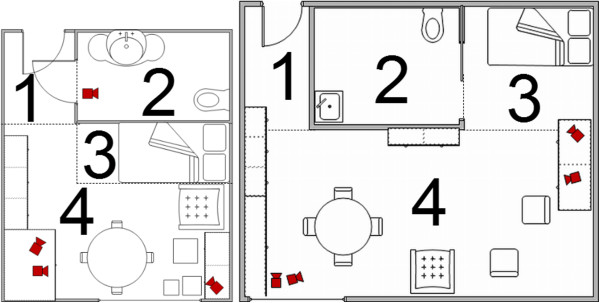
**Installation of FallCam camera system in participants’ rooms.** Right = assisted living room, left = residential care room 1 = hall, 2 = bathroom, 3 = sleeping area, 4 = living area.

The FallCam camera system was installed in each participant’s room, using a centralized PC platform in combination with standard IP cameras [13,14]. The assisted living rooms and residential care rooms consisted of two areas; one room with hall, living and sleeping area and a bathroom with toilet. Four cameras were placed to cover all areas of the room (Figure 1). Because of privacy issues, no cameras were installed in the bathroom except for one participant who fell repeatedly at that location. Before installation, a prospection visit was organized to inform participants and their relatives (if any) about the study and the camera system. Two to four weeks after the prospection visit and provision of written informed consent, the camera system was installed. All participants received weekly visits from two researchers, one with technical background to check the camera system (GD or JV), and one nurse with geriatric expertise (EV or MD) to fill out a standardized checklist on recent falls, overall health status and use of the camera system. Baseline assessment of functional status, mobility, physical performance, mood and cognitive function were ascertained. The Flemish version of the Triage Risk Screening Tool, a five-item multidimensional geriatric screening tool, was used to evaluate risk of functional decline [21]. Mobility was assessed using the Timed Up & Go Test [22]. Physical performance was measured with the Katz Index of Activities of Daily Living [23] and the Instrumental Activities of Daily Living [24], a six- and eight-item four-point Likert scale, respectively. Mood and cognitive status were ascertained using the ten-item Geriatric Depression Scale [25], and the Mini-Mental State Examination [26], respectively.

Image recordings (resolution 640 by 480 pixels, frame rate of 12 frames per second) were made 24 hours a day, seven days a week. After notification of a fall, video data of reported falls were saved starting from two hours before until two hours after the fall. A fall calendar with date and circumstances of the fall was completed by interviewing the person who fell and the supervising nurse if present.

### Analysis

Real-life images, with additional information from the fall calendar, nursing notes and medical charts, were used to analyze fall incidents. Two researchers (EV and MD) analyzed every fall independently, using the camera position with the clearest viewpoint. In case of doubt multiple camera positions were used. Disagreement was resolved through discussion with a third researcher (KM).

The classification system of Noury and colleagues [15] for reporting the process of falling was used to examine the phases of real-life falls by comparing the four phases of a fall with the real-life images. In line with Hauer et al. [27], falls were defined as *“an unexpected event in which the participant comes to rest on the ground, floor, or lower lever”* and classified into four phases. The first phase is the ‘prefall phase’, where the participant carries out any activity of daily living [15]. Fall characteristics analyzed in this phase were ‘activity before the fall’ (walking/transitioning on or off bed, sofa or chair/bending down/dressing/bathing/other) and ‘use of walking aids’ (yes/no). The ‘critical phase’ is described as “*an extremely short phase with a duration of 300–500 ms and consists of the sudden movement of the body toward the ground, ending with vertical shock on the ground”*[15]. During this second phase, the time of the fall (morning/noon/afternoon/evening/night), protective responses (yes/no), initial fall direction at the start of the fall (forward/sideward/backward/straight down), final landing configuration (forward/sideward/backward), perceived site of greatest energy absorption (head/pelvis, torso or buttocks/upper limb/lower limb) and impact to key body sites (head/pelvis/torso/hand or wrist/elbow or forearm/knee/shoulder), duration of the critical phase (in milliseconds) and presence of other persons (yes/no) was registered. In the third phase, ‘the postfall phase’, the participant is inactive, often lying on the ground [15]. Locations of the fall (living area/sleeping area/bathroom/hall/other), use of a call alarm (wireless personal call alarm or call alarm in the room) (yes/no), time needed to use the call alarm after a fall (in seconds), time of healthcare worker response (in minutes) and time on the ground (in minutes) were analyzed. During the last phase, ‘the recovery phase’, the participant gets up with or without help [15]. Fall characteristics examined were the ability to stand up unaided (yes/no) and fall-related injuries (none/minor/major). Major injury was defined as joint dislocation, fracture, and head injury that resulted in an emergency department visit or hospital admission [4]. Other injuries (e.g. bruises, sprains) were classified as minor injuries. Information about the type and severity of fall related injuries was collected by chart review, interview and observation. Analyses on protective responses (stepping and reach-to-grasp response), initial fall direction, landing configuration, perceived site of greatest energy absorption, and impact to key body sites were done based on the Fall Video Analysis Questionnaire (FVAQ) [28].

Descriptive analyses, including frequencies and percentages for the nominal/ordinal variables and means and standard deviation for the continuous variables, were conducted for all study variables. Those falls where a call alarm was or was not used were compared with the variable ‘time on the ground’ using the Mann–Whitney U-Test. Analyses were performed using SPSS version 17.0 for Windows (SPSS, Inc., Chicago, IL., USA).

### Ethical considerations

The study was approved by the Medical Ethics Committee of the Leuven University Hospitals, Belgium. All participants received oral and written information about the study and the FallCam camera system. A switch off button was installed in the room of every participant, allowing them to switch the system off at any time. During the study, none of the participants switched off the system. It was turned off sporadically by family or cleaning staff.

## Results

### Baseline characteristics of participants

A total of 17 months of data was collected. Participants were older females who lived alone in residential care facilities, had problems with mobility, ADL and IADL performance, used a walking aid, were at risk for falls and functional decline and took at least nine different drugs including drugs rated as high-risk for falls (e.g. psychotropics, antidepressants, diuretics). One person had cognitive impairment; none were at risk for depression (Table 1).

**Table 1:** Baseline characteristics of participants

**Characteristics**	**Participant A**	**Participant B**	**Participant C**
Age (years)	95	95	85
Place of residence	ALR	RCR	ALR
Hospitalization last 3 months	No	No	Yes
Number of drugs	13	12	9
Use of high-risk drugs^*^	Yes	Yes	Yes
Use of walking aid	Walking frame	Rollator	Rollator
Vision	Visually impaired	Glasses	Glasses
Hearing	Partly deaf	Hard of hearing	Hard of hearing
Fell in past year	Yes	Yes	Yes
TRST (range 0–5)	3	3	5
TUGT (sec.)	24	32	37
ADL (range 6–24)	10	13	16
IADL (range 8–32)	21	27	26
GDS (range 0–10)	3	3	1
MMSE (range 0–30)	28	27	16
Call alarm	CAR	CAR	PCA

ALR = assisted living room; RCR = residential care room; TRST = Flemish version of the Triage Risk Screening Tool (score ≥2 indicating at risk for functional decline)^19^; TUGT = Timed Up & Go Test (score ≥10 seconds indicating at risk for falls and fractures)^20^; ADL = Katz Index of Activities of Daily Living & IADL = Instrumental Activities of Daily Living (higher scores indicating more dependency)^21–22^; GDS = Geriatric Depression Scale (score >4 indicating at risk for depression)^23^; MMSE = Mini-Mental State Examination (score <24 indicating cognitive decline)^24^; CAR = call alarm in the room; PCA = wireless personal call alarm; ^*^Drugs rated as high-risk for falls e.g. psychotropics, antidepressants, diuretics.

### Characteristics of fall incidents

**Figure 2:**
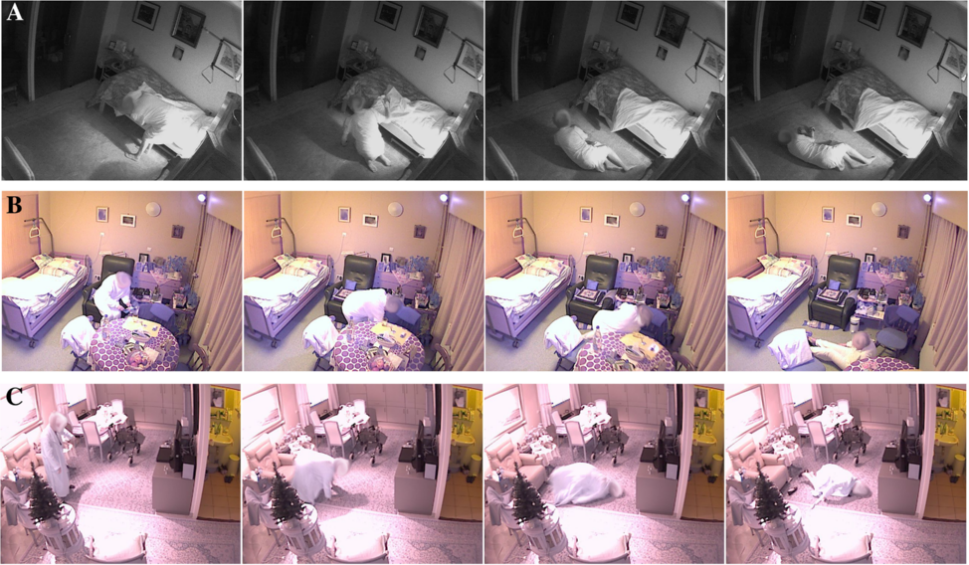
**Selection of video shots of fall incidents for each participant.** Participant **A**, Participant **B**, Participant **C**.

During the study period a total of 30 falls occurred of which 26 were recorded on camera (Table 2 and Figure 2). Two falls were not recorded due to technical problems and two because of their location (falls in a bathroom with no cameras installed).

**Table 2:** Fall characteristics

**Characteristics**	**Participant**			**Total**
	**A**	**B**	**C**	
Total number of falls	1	12	17	30
Number of falls on camera	1	10	15	26
Total camera time, months	5	7	5	17
*Prefall phase, n (%)*				
Activity before the fall				
*Transitioning*	0	2	7	9 (35)
*Walking*	0	2	5	7 (27)
*Bending down*	1	4	0	5 (19)
*Dressing*	0	1	3	4 (15)
*Bathing*	0	1	0	1 (4)
Use of walking aids	0	4	10	14 (54)
*Critical phase, n (%)*				
Initial fall direction				
*Backward*	0	5	9	14 (54)
*Sideward*	1	3	2	6 (23)
*Forward*	0	2	2	4 (15)
*Straight down*	0	0	2	2 (8)
Landing configuration				
*Backward*	0	5	11	16 (62)
*Sideward*	1	5	1	7 (27)
*Forward*	0	0	3	3 (11)
Perceived site of greatest energy absorption				
*Head*	0	0	0	0
*Pelvis/torso/buttocks*	1	9	11	21 (81)
*Upper limb*	0	1	2	3 (11)
*Lower limb*	0	0	2	2 (8)
Impact to key body sites				
*Head*	1	9	6	16 (62)
*Pelvis*	1	10	12	23 (89)
*Torso*	1	9	11	21 (81)
*Hand/wrist*	1	6	5	12 (46)
*Elbow/forearm*	1	8	7	16 (62)
*Knee*	0	1	5	6 (23)
*Shoulder*	1	8	3	12 (46)
Mean duration (± SD) of critical phase, in milliseconds^*^	2750^§^	1933 ± 538	1883 ± 719	1946 ± 639
Presence other persons	0	0	3	3 (11)
*Postfall phase, n (%)*				
Location fall				
*Living area*	0	6	11	17 (65)
*Sleeping area*	1	0	3	4 (15)
*Hall*	0	3	0	3 (12)
*Bathroom*	0	1	1	2 (8)
Use call alarm				
*Yes*	0	5	9	14 (54)
*No*	1	2	2	5 (19)
*No need°*	0	2	3	5 (19)
*Impossible to assess*	0	1	1	2 (8)
Mean time to use call alarm, sec.	NA^#^	34 ± 33	133 ± 56	70 ± 64
Mean time for healthcare worker response, min.	NA^#^	4 ± 3.9	9 ± 9.8	8 ± 8.4
Mean time on the ground, min.	30^§^	10 ± 13.7	15 ± 17.2	14 ± 15.8
*Unwitnessed falls - alarm used*	NA^#^	7 ± 4.6	13 ± 10.8	11 ± 9.2
*Unwitnessed falls - no alarm used*	30^§^	25 ± 30.4	30 ± 40.3	28 ± 25.4
*Recovery phase, n (%)*				
Stand up unaided	0	0	0	0
Fall-related injuries				
*None*	1	2	14	17 (65)
*Minor*	0	8	0	8 (31)
*Major*	0	0	1	1 (4)

Values are numbers (percentage) of falls captured on camera or mean ± SD; ^*^the mean duration of the critical phase was calculated for 21 falls; ^#^NA; not available because participant A only fell once and could not use the call alarm; °no need to use the call alarm because another person was in the room; ^§^standard deviation could not be calculated due to only one fall.

#### Prefall phase

Observed activities of daily living before the fall were transitioning on/off bed, sofa or wheelchair (n=9; 35%), walking (n=7; 27%), bending down (n=5; 19%), dressing (n=4; 15%) and bathing (performing personal hygiene at the sink) (n=1; 4%). Participant A fell only once, while bending down. Participant B (10 falls) fell mostly while bending down (n=4), while participant C (15 falls) typically fell while transitioning (n=7). In 14 (54%) falls the participants did use a rollator (n=11) or a wheel chair (n=3).

#### Critical phase

**Figure 3:**
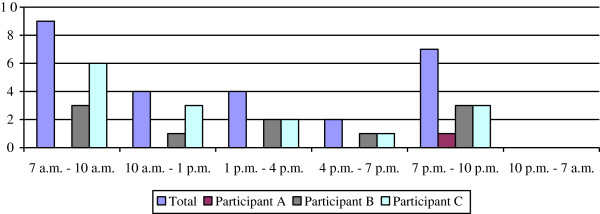
Time of fall incidents.

Figure 3 details the time of the falls. Sixteen falls (62%) occurred during the morning (7 a.m.-10 a.m.) or evening (7 p.m.-10 p.m.). No falls occurred at night. Participants mainly fell backwards: 14 falls had an initial primarily backwards fall direction (54%) and 16 had a final backwards landing configuration (62%). In 21 (81%) cases the perceived site of greatest energy absorption was the pelvis, torso or buttocks. The participants fell and impacted most frequently the pelvis (89%), torso (81%), head (62%), and elbow or forearm (62%).

In 21 falls (81%), the duration of the critical phase was calculated and on average lasted 1946 ms (SD=639, range 1075–3790). The other five falls (19%) consisted of two parts and duration was, therefore, not calculated: in three cases, residents B and C lost balance, but were able to hold on to an object (reach-to-grasp response) for a short time, and fell with a delay to the ground. In two other cases, another person was present during the fall and slowed down the event. In the majority of falls (n=23; 88%), however, no other persons were present.

#### Postfall phase

There were 17 (65%) falls in the living area, four (15%) in the sleeping area, three (12%) in the hall and two (8%) in the bathroom (Figure 1).

A call alarm (wireless personal call alarm or call alarm in the room) was used in 14 falls (54%) compared to 5 falls (19%) were the alarm was not used. In another five cases (19%) there was no need to call because another person was in the room and in two cases (8%) it was impossible to assess whether a call alarm had been used. When a call alarm was used, an average time of 70 seconds after the fall was needed to call for help (SD=64; range 15–224). Staff responded to the call after an average of eight minutes (SD=8.4; range 2–33).

Participants were lying on the ground for a mean time of 14 minutes (SD=15.8; range 2–59). In case a call alarm was used, the mean time on the ground was 11 minutes (SD=9.2; range 3–38), compared to 28 minutes (SD=25.4; range 2–59) when no call alarm was used and when no other person was in the room (Mann–Whitney U-test; p=0.445).

#### Recovery phase

None of the participants were able to get up unaided after a fall incident. In eight cases (31%) a fall resulted in a minor injury (e.g. hematomas or bruises). Only one major injury occurred, i.e. a laceration at the nose for which the participant was admitted to the emergency department.

### Examination of the phases of real-life falls

Activities before the fall (e.g. walking) could be clearly described in the prefall phase. Real-life images showed that the distinction between occasional sudden movements (e.g. lie down) and falls is essential for an effective fall detection system, as already suggested by Noury et al. [15]. Analysis of real-life images revealed that the critical phase of all falls lasted longer than 500 ms, in contrast to the classification system of Noury et al. [15]. In addition, protective responses were used: residents attempted to recover balance by stepping (stepping response) in 18 falls (69%) and in eight cases (31%) by reaching to grasp an object (reach-to-grasp response), and in six falls (23%) the resident rolled at the end of the critical phase, probably to reduce the impact shock with the floor. Whether the participant stayed inactive during the postfall phase depended on the individual. While participants A (one fall) and C (17 falls) remained inactive, participant B was mostly active (e.g. shuffling around on the floor, calling for help). The real-life fall risk images showed a recovery phase with residents standing up with help, as defined in the classification system.

## Discussion

This study provides new insights in the details of the fall process, based on a thorough analysis of 26 real-life fall events of three older persons with a high fall risk. The classification of Noury et al. [15] was used to describe the characteristics of the fall process during each fall phase.

Data from the prefall phase showed that most falls happened during walking or transitioning, consistent with previous studies [16,20,29,30]. Additionally, we found that in more than half of the falls a walking aid was used. This differs from Robinovitch et al. [20] who reported the use of walking aids in 21% of falls. They only included falls in common areas which could indicate more mobile and independent participants, as the participants in our study needed assistance to leave their room and were mobility aid users. Another possible explanation for this difference may be because of the small sample in this study (n=3, compared to n=130). Older people often have to use a walking aid because of impaired balance or an increased risk of falling. However, it has also been suggested that walking aids may be a risk factor for falls, because an increased attention for performing the dual-task is required [31]. While our data could support this suggestion, they could also be interpreted in support of the reasoning that frail older people who need walking aids tend to fall more.

Although most studies have not clearly defined the time period, the majority of falls happen during daytime hours [16,20,29,32], with patterns of physical activity contributing to a higher fall risk [33]. Consistent with these findings, participants in our study fell mainly during the day at times when they were most active.

Our results showed that participants fell backward in 62% of falls unlike other studies where participants mostly fell forwards [29] or sideways [34]. This difference might be due to the retrospective self-reportage with a risk of recall bias in those studies. Recall bias can influence outcomes, e.g. time on the ground may be over- or underestimated by the faller and the health care team [8,16]. Another possible explanation could be that the latter consisted of younger, community-living populations, with healthier participants. One study [33] with frail older persons in nursing homes showed a high level of backward falls similar to our study. Again, the small sample of participants with multiple falls made it difficult to draw any conclusion, as same fallers may tend to fall similarly in terms of fall direction. In addition, fall direction may be defined differently in literature, as the distinction between ‘initial fall direction’ and ‘landing configuration’ is not always clear [28].

Overall, the participants used their call alarm in 54% of the falls, after an average of 70 seconds. A prospective cohort study of 110 older persons residing in their own home, sheltered housing or in residential care [8] showed that 80% of individuals, who fell and had a call alarm, did not use their alarm. A distinction should be made between being unable to call because of practical reasons (e.g. not wearing wireless personal call alarm at the moment of the fall) or because of cognitive problems. Campbell [35] previously reported that activating the alarm may be prevented by impaired planning, coordination, and execution. In addition, Fleming et al. [8] detected a strong association between severe cognitive impairment and lying on the ground for a long time. Although we were unable to assess this association, participant C who was cognitively impaired needed much more time to use her personal call alarm - and as a consequence was lying on the ground for a longer time - as compared to participant B who was not cognitively impaired (Table 2). The difference in the mean time of lying on the ground with (11 minutes; n=14) compared to without a call alarm (28 minutes; n=5) was large, although not significant, which may be due to the small sample size.

Healthcare workers took an average of eight minutes to respond, with a range between two and 33 minutes. Although 33 minutes is a long time, it is probably not uncommon in health care settings, often characterized by low staffing levels. Healthcare workers frequently receive more than one alarm simultaneously without knowing which call is more urgent, or even which is because of a fall.

Time on the ground in the study of Fleming et al. [8] was substantially longer than in our study; 30% of fallers lay on the ground for over an hour. This difference might be explained because information about falls in Fleming’s study was gathered post-fall during a follow-up visit or phone call, leading to possible recall bias; and because of older persons living in different settings. Furthermore, lying on the ground for over an hour was less common in residential care facilities (8%) compared with participants living in sheltered housing (27%) or their own homes (13%). Despite these findings, even in residential care facilities, time on the ground is an important issue given its serious consequences [8].

All participants needed help to get up after a fall. These findings are in contrast again with studies from younger, community living persons where only 20 to 43% were unable to get up after a fall [8-10]. Fleming et al. [8] reported that all participants living in institutionalized care needed help to get up. This challenging matter underlines the importance of fall prevention and regular surveillance of older persons with a high risk of falling.

Overall, our study results were consistent with the classification system by Noury et al. [15]. The real falls corresponded well with the prefall and recovery phase. For the critical phase, our data showed a prolonged critical phase in all falls. This might be due to the fact that the critical phase can be decelerated as deceleration and a prolonged reaction-time is a common feature of older persons [36]. With regard to the end of the critical phase, i.e. the phase where the body normally hits the ground or an obstacle, attention must be paid to the protective measure of rolling because the ‘impact shock’ [15] may be reduced by this movement. Based on our findings, we would suggest distinguishing two separate phases in the critical phase. First, a prolonged loss of balance phase including attempts to balance recovery with protective responses such as stepping and second, the actual descending phase after failure to recover balance in which the vertical velocity increases linearly with time because of gravitational acceleration, resulting in the impact of the body on the ground. In addition, there is a different type of fall, where the fall consists of two parts, e.g. the resident starts to fall but is able to hold on to an object or another person is able to slow down the event and there is a delay in the individual touching the ground. In our study, the postfall phase was not characterized by inactive participants. Being active or inactive depended on the individual. Further research for developing and testing fall detection systems should take these findings into account.

This study was the first to analyze fall incidents in older people’s person living space by means of video images in a real-life environment. One other study measured falls with video cameras in common areas of long-term care facilities (e.g. dining rooms, lounges, hallways) [20], representing a different environmental and situational context. Although a comparison between both studies is difficult due to the small sample in our study, falls in common areas mostly happened during walking forward while falls in private areas happened mostly during transitioning. Mobility aids were used twice as often in private area falls compared to falls in common areas. The majority of studies [8,10,29] used self-reported and/or retrospective methods for collecting falls data, which makes the ascertainment of circumstances less reliable. Furthermore, several previous studies used fall simulations to describe the fall process and evaluate fall detection systems. These simulations were mostly uncomplicated falls performed by healthy, young volunteers and not by older persons with an actual risk of falling [37]. Several other studies [38-40] collected fall-related data from real-life falls, using accelerometers and/or infra-red sensors. However, the precise fall process was unknown because image recording was not available. As stated by Bagalà et al. [41], testing fall detection systems in real-life conditions is essential to produce more effective automated alarm systems with fewer false alarms and a higher acceptance. Indeed, our study results give more insight into the complexities of the fall process that should be considered in designing and testing of fall detection algorithms (e.g. most common sequence of events, such as activities leading to falls, and subsequent causes of imbalance).

Although not the aim of our study, another implication is its importance for educational purposes. While analyzing, it became clear that our data were particularly relevant for clinical practice to increase awareness of behavioral and environmental factors causing falls. For example, participant B fell three times in similar circumstances, taking clothes out of a bottom drawer of a closet, suggesting that a rearrangement of the closet might prevent future falls. Another example was the low awareness of staff of possible fall causes (e.g. guiding the participant to bed and forgetting to put the walking aid in the vicinity). The researchers felt ethically obliged to inform staff and management of the facility. At the end of the study, a feedback session was held to inform and educate staff and management of the facility. Video data with modifiable circumstances were presented to discuss falls and fall prevention, after additional approval had been obtained from the participants.

Our study has some limitations. Although 26 falls were recorded, only three older, female residents participated in the study, and of those, one resident only fell once. Despite efforts to enhance participation (e.g. recruitment via staff because of the confidential relationship with residents), the participation rate was low, possibly due to the profound nature of the study, and probably resulted in selection bias. For example, two of the residents had a high MMSE score not representative for the general population in ALR and RCR where most people have impaired cognitive function. Hence, our findings may not be generalizable. However, fall characteristics were explored and a better understanding of the fall process was obtained. Another limitation was that falls outside the rooms of the participants could not be detected, as the FallCam system was restricted to this area. Finally, ethical issues should be mentioned. The use of cameras to detect falls can raise ethical concerns (in particular with regard to privacy). The select group of participants in this study accepted the installation and use of the camera system, but there was some initial hesitation during the recruitment period. Similarly, a British community survey [42] reported that privacy concerns hampered the acceptance of automatic fall detection units. Therefore, further qualitative research is needed to understand older people’s perceptions of the acceptability of this type of surveillance technology before more future investment in the technical development.

During the feedback session at the end of the study, group discussions were held to learn more concerning the perceptions of staff. Overall, staff viewed the technology as positive and thought that a full operational camera system might help their work. The main reasons why the camera system could contribute to the care of older persons were found to be: sense of security for residents, the ability to provide rapid assistance in case of a fall and preventing lying on the floor for a long time after falling. However, technology can only ever be a tool to improve care, not a substitute for the need for skilled and caring people providing care in person.

## Conclusions

The development of fall detector systems that can accurately identify falls and alarm accordingly has potential to reduce harmful “long lies” on the floor, but the reliability of such technology depends on detection algorithms reflecting the reality of fall patterns in the population affected. This study contributes to a better understanding of the complexities of the fall process in older persons with a high risk of fall. Further research with a larger and more representative sample is needed to confirm our findings.

## Competing interests

This study was funded by the Institute for the Promotion of Innovation through Science and Technology in Flanders (IWT-Flanders), Belgium.

## Authors’ contributions

All authors participated in the design of the study. EV, MD and GD contributed to the recruitment of participants and carried out the data collection. EV, MD, ED, KM were responsible for the data interpretation and analysis. EV drafted the manuscript. MD, GD, TG, BV, ED, SB, KM carried out the critical revision of the manuscript. Supervision was done by KM. All authors contributed to revisions and approved the final manuscript.

## Pre-publication history

The pre-publication history for this paper can be accessed here:

http://www.biomedcentral.com/1471-2318/13/103/prepub
